# Performance Profiles: A New Approach Based on Training Focused on Physical Aspects Rather than Technical–Tactical Ones

**DOI:** 10.3390/sports13110402

**Published:** 2025-11-10

**Authors:** Amalia Campos-Redondo, Almudena Martínez-Sánchez, Pablo López-Sierra, Eduardo Chacón-Fernández, Javier García-Rubio

**Affiliations:** Optimization of Training and Sports Performance Research Group (GOERD), Faculty of Sports Science, University of Extremadura, 10003 Cáceres, Spain; amcamposr@alumnos.unex.es (A.C.-R.); almudenams@unex.es (A.M.-S.); pablols@unex.es (P.L.-S.); ecnfernandez@gmail.com (E.C.-F.)

**Keywords:** football, performance profiles, GPS

## Abstract

This study aimed to identify distinct external load profiles of 23 semi-professional football players (22.52 ± 1.74 years) during four official matches (40 cases in total; 10 per match). Using GPS-based inertial technology WIMU PRO (Hudl, Lincoln, NE, USA), data were collected to analyze players’ physical performance. A principal component analysis (PCA) identified three performance profiles—“Total Player,” “Explosive Player,” and “Dynamic Player”—that together explained 70.08% of the variance. These profiles revealed that players may share similar physical characteristics despite occupying different on-field positions. Training players based on their physical performance profiles, rather than solely on their tactical roles, may enhance both individual development and overall team performance. This approach offers a novel framework for individualized conditioning in team sports.

## 1. Introduction

Performance analysis has become a fundamental tool for researchers, sports organizations, clubs, athletes, and coaches [1]. Understanding, therefore, the physical demands required in a football match is essential for coaches and fitness trainers to properly prepare and plan a training program. This ensures the improvement of players’ performance [2,3].

For many years, a wide variety of tools have been used to define the technical–tactical performance profiles of football players. Recently, GPS technology has been added to the techniques used for the monitoring of training sessions and competitions, providing valid and reliable measures associated with players’ physical profiles [4,5]. The use of this technology enhances the understanding of players’ performance profiles and how these profiles evolve according to age, specific field positions, and the context of competition and training [6,7]. GPS devices have been employed by numerous football clubs to monitor players’ training load, yet few studies have applied this technology to gain insight into players’ physical profiles during official competitive matches [8].

From the outset, it has been maintained that the 11 players who make up a football team must assume specific roles and demands based on their position on the field to achieve success. While players and coaching staff have a universal understanding of the technical, tactical, and physical components required for each position, there is limited scientific information to support these aspects [9]. Several articles have suggested the key characteristics that may influence player performance in specific positions [10]. Although several studies have analyzed the relationship between players’ positions and their physical demands, much of this research relies on subjective evaluations from coaches rather than objective data [9].

Consequently, the information reported is often inconsistent and lacks consensus across studies. This highlights the need for objective analyses based on quantitative performance data to better understand how physical demands differ among players and positions [11,12,13]. Additionally, football players have been categorized into positions such as goalkeeper, central defender, full-back, central midfielder, wide midfielder, and forward [14]. For instance, midfielders tend to cover nearly twice the high-intensity running distance compared with central defenders [15]. Consequently, training programs should be designed to reflect these positional differences. Moreover, it is well established that a player’s tactical role is a major determinant of their match-related physical performance. Therefore, conditioning stimuli must account for the tactical context to ensure an optimal transfer of training adaptations to match performance [16,17]. Incorporating this understanding into training design is essential to bridge the gap between physical preparation and the real demands of competition.

Recent studies have analyzed the influence of external training loads within weekly training sessions in football [14]. However, most of this research has been conducted in professional contexts and focused primarily on training microcycles, providing limited insight into how these loads translate to actual competition, particularly in semi-professional or amateur settings. Moreover, few studies have examined the classification of players based solely on their physical performance profiles, independently of their technical–tactical roles or positional labels. Addressing this gap, the main objective of the present study was to identify different external load profiles of players from the same team during four official matches of a semi-professional football team. Specifically, the study aimed to group players according to these profiles and determine the relationship between their on-field positions and the physical demands associated with their performance profile.

## 2. Materials and Methods

### 2.1. Study Design

The research design employed in this study is observational, within the framework of a descriptive strategy, as data collection is carried out in an ecological manner [18]. Furthermore, it is classified as ex post facto with a multivariate approach, as the variables are analyzed simultaneously to understand their complex relationships [19].

### 2.2. Participants

A total of 23 outfield players (22.52 ± 1.74 years) from a team competing in the Tercera División RFEF category participated in this study during four official season matches. The final analysis included 40 valid cases (10 per match), corresponding exclusively to players who completed the full duration of the game without substitutions. The mean playing time across all analyzed players was 90 ± 3.2 min, confirming that only complete match performances were considered to ensure data consistency. A non-probability convenience sampling method was used, as participants were selected based on availability, proximity, willingness, and ease of access.

The reality of sports training is that the number of players analyzed is relatively low, due to the competition context. This reality of sports context does not limit the validity of its results [20,21]. Also, the four games analyzed were selected because the study was conducted during a competitive microcycle in which the team played against direct rivals of comparable quality and objectives. All matches were carried out under a consistent tactical framework defined by high pressing, man-to-man defensive strategies, and fast counterattacks. This homogeneity in competitive and tactical context minimized external variability and strengthened the reliability of the physical performance profiles identified. Nevertheless, the findings should be interpreted with caution due to the limited sample size, which restricts generalization beyond this specific context.

All team members, along with the coaching staff, signed an informed consent form, agreeing to the use of the data collected, analyzed, and compared, as well as being informed of the study’s advantages and potential risks. This research adhered to the criteria outlined in the Declaration of [22]. Table 1 shows anthropometric data of the sample.

The variables in this study, which were extracted and normalized to each player’s playing time, are shown in Table 2.

### 2.3. Instruments

**Figure 1:**
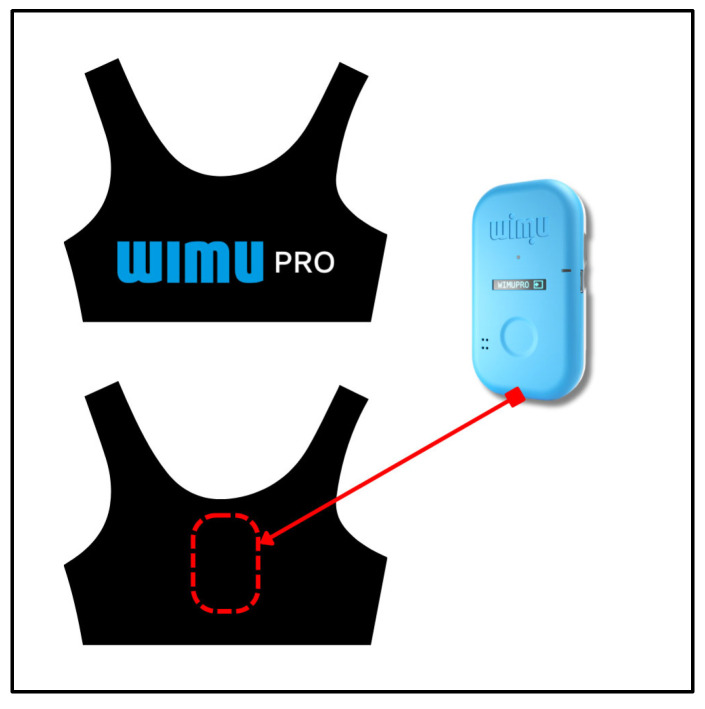
Equipment used for data collection.

For data collection in this research, the following instruments were used: (i) WIMU PRO (from Hudl, NE, USA), an inertial device that incorporates four accelerometers in three dimensions, in addition to other sensors such as gyroscopes, a global positioning system (GPS), and Ultra-Wideband (UWB) technology [23] (ii) Inertial device harness, which securely holds the device in place via a fixed pocket located between the scapulae. Figure 1 shows the material used for the attachment of the inertial device.

The players were informed in advance about the equipment that would be provided for each of the four matches. Additionally, a familiarization process with the equipment used for the measurements was conducted during a training session prior to the matches. For all four matches, a protocol was established in which the players were equipped with specific vests that carried a WIMU inertial device before the warm-up. To differentiate the match phases—such as the warm-up, first half, and second half—the SVIVO application (RealTrack Systems, Almería, Spain) was used, which allowed the segmentation of the phases for easier data analysis later on.

### 2.4. Statistical Analysis

The statistical analysis was conducted using Jamovi 2.3.28 software (The Jamovi Project, v.2.3, 2022). A factorial analysis model (principal component analysis, PCA) was used to reduce the 20 variables related to selected physical characteristics into dimensions or factors. The model was established with Varimax rotation adjustment, interpreting the rotated matrix with coefficients greater than 0.6, and an eigenvalue of 1.5 was set to determine the dimensions. That is, factors were selected based on eigenvalues greater than this threshold. During this analysis process, performance indicators with higher factor loadings were identified. This methodology allows for grouping performance indicators into fewer factors that reflect different playing styles, thereby facilitating the identification of player profiles based on their performance on the field [24].

## 3. Results

Table 3 shows the results of the factorial analysis (principal component analysis). The data indicated a statistically significant model (chi-square value = 948; *p* < 0.001; KMO = 0.603), identifying three components that explain 70.08% of the variance. The KMO value and the significance of Bartlett’s test confirmed the adequacy of the sample and the validity of applying the principal component analysis (PCA) to identify the underlying performance dimensions. The factor loadings indicate how each variable correlates with the principal components. Based on this, the model revealed three dimensions, which are distributed as follows:

(i) Dimension 1 (“Total Player”): Eigenvalue of 6.96, explained variance of 34.8%. This component seems to generally represent the volume of activity and intensity of the players’ effort. The variables with high loadings in this component include total distance covered, player activity at different speed ranges, as well as decelerations and the load endured by the player during the match. Therefore, this component can be interpreted as an index of the players’ overall activity.
(ii) Dimension 2 (“Explosive Player”): Eigenvalue of 5.09, explained variance of 25.45%, with the following variables within this dimension: [24.00–50.00] km/h; Maximum Acceleration (m/s^2^) (0.661); Maximum Speed (km/h) (0.796); [24.00–50.00] km/h Count (0.852); Maximum Sprint Count (0.821). This component appears to be related to high-intensity actions. The variables with significant loadings in this dimension are distance covered at speeds over 21 km/h, maximum speed, and sprints.
(iii) Dimension 3 (“Dynamic Player”): Eigenvalue of 2.11, explained variance of 10.55%, with the following variables within this dimension: Accelerations; Decelerations. This component focuses on accelerations and decelerations, which can be interpreted as changes in pace that occur during the game, specifically the frequency of speed changes.

The eigenvalues from the principal component analysis are shown in Table 4. Additionally, each eigenvalue indicates the amount of variance in the data explained by each principal component. In this way, Component 1 explains 34.83% of the total variance in the data, making it the most significant component in terms of explained variance. Component 2 explains 25.46% of the variance, and finally, Component 3 explains 10.55%, bringing the cumulative percentage to 70.8%.

**Figure 2:**
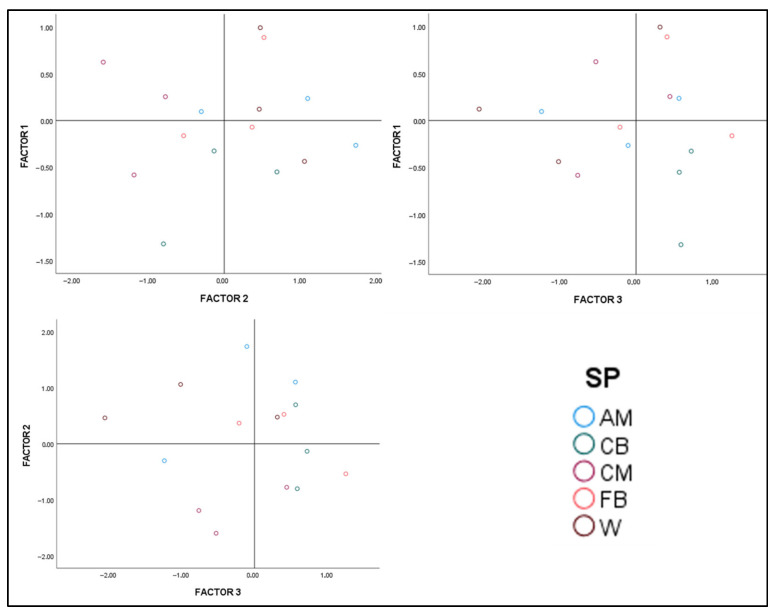
Graph of specific team positions grouped into three performance profiles. The score on each axis indicates membership in that factor (if positive). Abbreviations: CM = Central Midfielder; AM = Attacking Midfielder; FB = Full Back; CB = Center Back; W = Winger.

Figure 2 illustrates the playing styles based on physical demands according to different profiles.

## 4. Discussion

The main objective of the present study was to identify different external and internal load profiles of football players during four official matches of a semi-professional team. After a data analysis process, the results revealed three distinct physical performance profiles among the team’s players, based on the load they experienced during the four matches. Training players based on their physical performance profiles rather than their specific positions represents an innovative approach that could optimize the development of individual potential and team performance. The study focused exclusively on competition data to isolate the physical demands experienced during official matches, without including training loads, which would reflect a different component of players’ preparation.

The evolution of the sport highlights the need to align with what occurs in competition, which can be achieved by monitoring various quantitative game parameters [25]. Therefore, defining these physical profiles can be of great importance when analyzing performance data. In fact, some studies have provided different perspectives on the influence of physical condition on a team’s success, specifically focusing on the relationship between physical performance and game statistics with the number of points achieved by the team [26]. In this case, the analysis allows for an understanding of a player’s score in each of the three factors, which determines their reliance on specific actions [24]. Currently, studies often examine player performance profiles based on technical–tactical characteristics or specific positions [27,28]. Thanks to the results obtained from this research, it can be observed that, within the same position (attacking midfielder, winger, central midfielder, etc.), players can have performance profiles with very different physical characteristics. This indicates that not all players, despite playing in the same position, necessarily have to perform the same role on the field or possess the same physical attributes.

Dimension 1 (“Total Player”) is defined by variables related to the total distance covered across different speed zones and the number of high-intensity decelerations. This component reflects players who sustain a high physical load and maintain consistent activity throughout the match, combining aerobic endurance with repeated efforts at moderate and high intensities. These findings are consistent with previous research demonstrating that central midfielders typically cover the greatest total distances and perform the highest number of moderate- to high-intensity running bouts, reflecting their role in linking defensive and offensive phases of play [29,30]. Similarly, studies have shown that players occupying central positions exhibit high workloads due to their continuous participation in both transition and possession phases [28,31]. Furthermore, research highlighted that midfielders maintain performance across the match despite fatigue, evidencing greater aerobic endurance and recovery capacity compared to other positions [30].

Dimension 2 (“Explosive Player”) reflects the ability to perform short, high-intensity efforts involving rapid accelerations, sprints, and maximal speed actions, relying on anaerobic power and neuromuscular explosiveness to create decisive situations during match play.

The strong loading of variables such as maximum speed, number of sprints, and distance covered above 24 km/h indicates that this dimension distinguishes players capable of repeated maximal efforts over short durations. These findings align with previous research showing that forwards and wingers reach the highest peak speeds and sprint frequencies during competition, reflecting their tactical roles in offensive transitions and space creation [26,27]. Similarly, several studies highlighted that players occupying wide areas—such as wingers and full-backs—are often involved in repeated sprinting actions, particularly in teams adopting counter-attacking styles [15,28].

In the present study, wingers showed the highest scores for this component, confirming their reliance on speed and acceleration to create numerical advantages on the flanks [15]. However, players from other positions, such as full-backs and attacking midfielders, also showed high scores, suggesting that explosive capabilities are not exclusive to wide players [32]. This supports the notion that modern football demands high-intensity physical attributes across different tactical roles, promoting greater flexibility and adaptability [33,34].

Dimension 3 (“dynamic player”) is primarily composed of players who excel in accelerations and decelerations. These variables indicate the players’ ability to perform short, high-intensity efforts, which are crucial in game situations that require actions such as changes in pace. These actions fall within the specific movement qualities in football, provided they adapt to interactions with the environment, opponents, and teammates [35]. Central defenders score highly in this dimension, as despite not typically covering long distances at high intensity [36], they need to make continuous adjustments to their pace. Additionally, central midfielders, full-backs, and attacking midfielders also show high scores in this profile, supporting the idea that accelerations and decelerations are key performance actions across multiple positions. These abilities contribute significantly to overall player performance and should be specifically developed across all playing roles [37].

As can be seen in the graph of factors 2 and 3, attacking midfielders score positively in several quadrants; some exhibit high levels of explosiveness, while others display a more standard profile. This information highlights that, within this team, the physical attributes of attacking midfielders are not homogeneous, which may challenge the idea of training them in the same way. Additionally, central midfielders also show variability, with some possessing qualities that reflect intensity and endurance, while others exhibit a less physically demanding profile, potentially being more strategic and technical in nature.

The highly competitive demand makes it essential for professionals such as physical trainers and sport scientists to monitor variables that provide information on training load and the physical characteristics of players [38]. The study of the relationships between individuals and variables could help in planning a type of training that is adapted and tailored to the players, with the aim of optimizing performance [39]. For this reason, training players based on their physical performance profile allows for a comprehensive development of their natural abilities and maximizes their individual performance. In fact, it is likely that the impact of each player on the team will increase, as they will not be restricted by the specific demands of their position. While a professional player’s technical and tactical qualities determine their performance in the game, optimal physical fitness enables them to perform a wide range of activities and remain actively involved in the game [40]. In relation to this, several studies have demonstrated that when fatigue sets in, players tend to disengage from the ball and the game [41].

Recent evidence suggests that classifying players solely according to their tactical or positional roles may overlook important differences in physical performance profiles. Several authors have argued that the increasing physical and physiological demands of modern football require a more individualized approach to player monitoring and training [37,42]. Grouping athletes based on their physical load characteristics—such as acceleration, deceleration, sprinting ability, and endurance—can provide a more objective framework for optimizing training adaptations and reducing injury risk [43]. It has been highlighted in several studies that players with similar physical profiles often fulfill comparable physical functions across different tactical systems, regardless of their nominal position on the field. Therefore, organizing players according to their physical performance roles rather than their tactical labels may enhance the specificity and efficiency of conditioning programs in team sports [44].

Moreover, this training approach can help the team achieve greater tactical flexibility, as players will be able to adapt to different positions depending on the context and demands of the match, promoting the development of versatile players. Some authors concluded that the style of play did not significantly alter the physical demands placed on players [45]; however, other studies have shown contradictory conclusions [32]. In fact, data from the team shows that a full-back with high speeds and agility can take on the role of a winger, if necessary, as they share a similar physical profile.

Following the conclusions drawn by several authors, it can be argued that “traditional football,” based solely on a player’s talent in a specific position, has been replaced by a more versatile style of football, where players assume multiple roles. This intensification of the game has begun to emerge primarily due to the dynamic physical capabilities of players, which can increase tactical options during matches. This highlights the need to analyze the latest research in the field of physical training in football to identify emerging trends [46,47].

The main limitation of this study lies in its small and context-specific sample, composed of 23 players from a single semi-professional team, which limits the generalization of the findings. However, data were collected during a competitive microcycle against direct rivals of similar level, under a consistent tactical framework based on high pressing, man-to-man defense, and fast counterattacks, ensuring internal consistency and ecological validity. A major strength of the study is the application of a multivariate approach to identify physical performance profiles independently of players’ technical–tactical roles. Future research should extend this methodology to larger samples, different competitive levels, and alternative tactical models to confirm and expand the current findings.

## 5. Conclusions

The main objective of this study was to identify different external and internal load profiles of players from the same semi-professional football team during four official matches. The results revealed the presence of distinct physical performance profiles among players, regardless of their technical–tactical roles or on-field positions. This indicates that some players share similar physical demands despite fulfilling different positional responsibilities.

These findings suggest that training programs could potentially benefit from being adapted according to players’ physical performance profiles rather than exclusively based on their playing positions. However, this should be understood as a practical implication derived from the observed data, not as an empirically verified outcome. The study provides a methodological framework for grouping players according to physical characteristics observed in competition and offers a new perspective that may guide future research on individualized conditioning in team sports.

Future studies should test the effectiveness of this approach in real training and competitive contexts, comparing profile-based conditioning with traditional position-based models to determine its actual impact on player performance.

## Figures and Tables

**Table 1:** Anthropometric data of the subjects.

Subject	Specific Position	Weight (kg)	Height (m)
1	Central Midfielder	74.80	1.80
2	Center Back	74.10	1.79
3	Attacking Midfielder	68.00	1.72
4	Winger	7500	1.83
5	Forward	73.50	1.79
6	Central Midfielder	59.30	1.65
7	Center Back	88.00	1.84
8	Attacking Midfielder	61.70	1.69
9	Winger	72.20	1.76
10	Full Back	74.70	1.75
11	Full Back	67.10	1.67
12	Forward	76.20	1.85
13	Full Back	62.50	1.67
14	Central Midfielder	52.00	1.69
15	Central Midfielder	70.00	1.76
16	Central Midfielder	67.80	1.82
17	Center Back	72.50	1.82
18	Winger	64.40	1.77
19	Central Midfielder	68.30	1.78
20	Forward	65.50	1.71
21	Attacking Midfielder	70.00	1.78
22	Winger	66.00	1.73
23	Winger	52.20	1.73

**Table 2:** Dependent variables used in the study.

Variables	Unit	Definition
Distance	Meters (m)	Total distance covered by the player during match play
Explosive distance	Meters (m)	Total distance covered while accelerating above 1.12 m·s^−2^.
[60.00–12.00] km/h	Meters (m)	Total distance covered within this absolute speed zone (low-intensity running).
[12.00–18.00] km/h	Meters (m)	Total distance covered within this absolute speed zone (moderate-intensity running)
[18.00–21.00] km/h	Meters (m)	Total distance covered within this absolute speed zone (high-intensity running).
[21.00–24.00] km/h	Meters (m)	Total distance covered within this absolute speed zone (very-high-intensity running)
[21.00–24.00] km/h	Count (n)	Number of times the player entered the 21.00–24.00 km/h speed zone.
[24.00–50.00] km/h	Meters (m)	Total distance covered within this absolute speed zone (sprinting).
[24.00–50.00] km/h	Count (n)	Number of times the player entered the 24.00–50.00 km/h speed zone.
Accelerations	Count (n)	Total number of accelerations greater than 3 m·s^−2^.
Decelerations	Count (n)	Total number of decelerations lower than −3 m·s^−2^.
Maximum acceleration	m/s^2^	Highest acceleration value recorded during the match.
Maximum deceleration	m/s^2^	Highest deceleration value recorded during the match.
High-intensity accelerations	Count (n)	Number of accelerations performed above 3 m·s^−2^.
High-intensity decelerations	Count (n)	Number of decelerations performed below −3 m·s^−2^.
Maximum speed	Km/h	Peak running speed reached by the player.
Maximum sprint	Count (n)	Number of sprints performed above the sprint threshold (18.80 km/h).
Total impacts	Count (n)	Total number of body impacts recorded by the inertial device.
High-intensity impacts	Count (n)	Number of impacts exceeding 8 G-force
Player Load	a.u	Composite measure of the total mechanical load experienced by the player during the match.

**Table 3:** Principal component loadings.

Variables	1	2	3
Explosive Distance (m)	0.936		
[18.00–21.00] km/h (m)	0.853		
[12.00–18.00] km/h (m)	0.819		
Distance (m)	0.803		
High-Intensity Decelerations Count	0.732		
Player Load	0.729		
[21.00–24.00] km/h Count	0.720		
[21.00–24.00] km/h (m)	0.665	0.646	
Total Impacts Count	0.656		
[6.00–12.00] km/h (m)	0.604		
High-Intensity Accelerations Count			
High-Intensity Impacts Count			
[24.00–50.00] km/h (m)		0.886	
Maximum Speed (km/h)		0.843	
Maximum Sprint Count		0.832	
[24.00–50.00] km/h Count		0.822	
MAX Acceleration (m/s^2^)		0.686	
Decelerations			0.924
Accelerations			0.922
MAX Deceleration (m/s^2^)			

Note: Each component represents a different physical performance dimension identified through principal component analysis (PCA).

**Table 4:** Initial eigenvalues.

Component	Eigenvalue	% of Variance	Cumulative %
1	6.9668	34.83395	34.8
2	5.0915	25.45775	60.3
3	2.1098	10.54906	70.8
4	1.3821	6.91069	77.8
5	1.0450	5.22490	83.0
6	0.8531	4.26533	87.2
7	0.5770	2.88518	90.1
8	0.4681	2.34056	92.5
9	0.3950	1.97521	94.4
10	0.2947	1.47325	95.9
11	0.2025	1.01251	96.9
12	0.1852	0.92613	97.9
13	0.1350	0.67494	98.5
14	0.0862	0.43095	99.0
15	0.0763	0.38141	99.3
16	0.0474	0.23704	99.6
17	0.0407	0.20325	99.8
18	0.0315	0.15767	99.9
19	0.0116	0.05791	100.0
20	4.63 × 10^−4^	0.00231	100.0

## Data Availability

The data presented in this study are available on request from the corresponding author due to privacy restrictions.
